# Magnetization switching of a perpendicular nanomagnet induced by vertical nonlocal injection of pure spin current

**DOI:** 10.1038/s41598-019-56082-x

**Published:** 2019-12-20

**Authors:** Hirofumi Suto, Tazumi Nagasawa, Taro Kanao, Kenichiro Yamada, Koichi Mizushima

**Affiliations:** Corporate Research and Development Center, Toshiba Corporation, 1, Komukai-Toshiba-cho, Saiwai-ku, Kawasaki 212-8582 Japan

**Keywords:** Spintronics, Magnetic devices

## Abstract

Injection of pure spin current using a nonlocal geometry is a promising method for controlling magnetization in spintronic devices from the viewpoints of increasing freedom in device structure and avoiding problems related to charge current. Here, we report an experimental demonstration of magnetization switching of a perpendicular magnetic nanodot induced by vertical injection of pure spin current from a spin polarizer with perpendicular magnetization. In comparison with direct spin injection, the current amplitude required for magnetization switching is of the same order and shows smaller asymmetry between parallel-to-antiparallel and antiparallel-to-parallel switching. Simulation of spin accumulation reveals that, in the case of nonlocal spin injection, the spin torque is symmetric between the parallel and antiparallel configuration because current flows through only the spin polarizer, not the magnetic nanodot. This characteristic of nonlocal spin injection is the origin of the smaller asymmetry of the switching current and can be advantageous in spintronic applications.

## Introduction

Injection of spin current into a magnetic material can induce magnetization switching and magnetization oscillation, which are the operating principle of spintronic devices such as magnetic tunnel junctions (MTJs) explored for magnetoresistive random-access memory (MRAM) and spin-torque oscillators (STOs) explored for microwave-assisted magnetic recording (MAMR)^1^. The simplest form of spin injection is realized by applying a charge current through a magnetic/non-magnetic/magnetic trilayer structure. Recently, injection of pure spin current using a nonlocal geometry has been extensively studied as another method of spin injection^2–6^, and magnetization switching^3–5^ and magnetization oscillation^6^ of in-plane magnetization have been demonstrated. In this method, the charge current and spin current paths are separated, and only the spin current is introduced to the active magnetic layer, while the charge current bypasses it. This characteristic increases freedom in device structure and frees the active magnetic layer from problems related to the charge current, such as heat generation and Oersted fields. Owing to these advantages, nonlocal spin injection can realize a new type of MTJ and STO, which we discuss at the end of this section in comparison with the sample used in this study. Nonlocal spin injection has also been explored for the read sensor in magnetic recording, which can increase spatial resolution by placing the pinned layer stack outside of the gap area and reducing the shield gap^7^.

Nonlocal spin injection is comparable to spin-orbit torque (SOT) in that both methods can generate a spin current unaccompanied by a charge current. SOT has also attracted attention, and SOT-based magnetization switching, magnetization oscillation, and domain wall motion have been reported^8–20^. One advantage of nonlocal spin injection over SOT is that low-resistivity materials such as Cu, Ag, or Al have good spin transport properties. Namely, when a current is applied through a spin polarizer and non-magnetic layer composed of the above materials to inject spin, the non-magnetic layer also works as a low-resistivity electrode. On the other hand, efficient SOT materials such as Ta, Pt, W, Ir or topological insulator have high resistivity, which increases power consumption or requires an additional electrode to carry current to the SOT material. In addition, contact with efficient SOT materials can enhance the damping constant of the active magnetic layer, increasing current required for the operation^21^.

Besides the resistivity and damping issue, nonlocal spin injection has more freedom in the direction of the injected spin. SOT injects only spin having direction parallel to the magnetic layer/SOT layer interface. Because of this restriction, SOT cannot induce switching of perpendicular magnetization or out-of-plane magnetization oscillation unless an external field^9–12^, anisotropy gradient^13^, additional magnetic layers^14–17^, or an additional spin source^18^ are employed. In nonlocal spin injection, spin with direction perpendicular to the magnetic layer/non-magnetic layer interface can be injected by using a perpendicular magnetic material in the spin polarizer. This kind of spin injection solely realizes switching of perpendicular magnetization and out-of-plane magnetization oscillation, which are the key technologies for realizing high-density MRAM and MAMR, respectively^22–26^. However, research studies on non-local spin injection have been limited to in-plane magnetization.

**Figure 1:**
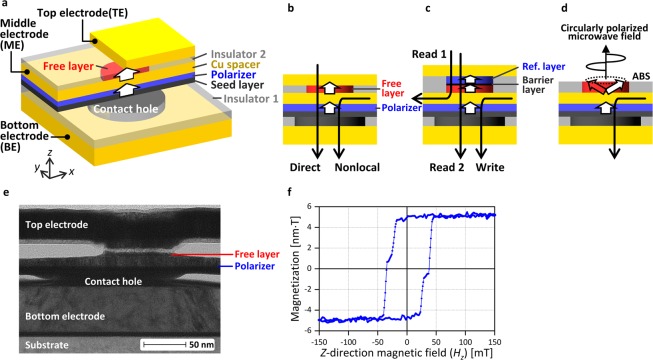
Sample structure and magnetic property of the GMR film. (**a**) Three-dimensional and (**b**) side-view schematic of the sample structure. (**c**) Three-terminal MTJ based on the sample shown in (**a**). (**d**) STO for MAMR based on the sample shown in (**a**). (**e**) Cross-sectional transmission electron microscopy image of the sample. (**f**) Perpendicular magnetization loop of the GMR film measured by using VSM.

In this paper, we demonstrate magnetization switching induced by pure spin current using a vertical nonlocal spin injection structure with a perpendicular active magnetic layer (free layer) and a perpendicular spin polarizer. The schematics of the sample are shown in Fig. 1a,b. This vertical nonlocal structure is beneficial from the viewpoint of scalability of the device because spin injection is performed from beneath the active magnetic layer and does not require additional lateral space. The sample structure enables both nonlocal and direct spin injection, as shown in Fig. 1b. In comparison with direct spin injection, nonlocal spin injection induces magnetization switching at a similar current amplitude, and shows smaller asymmetry of the switching current (*I*_sw_) between parallel-to-antiparallel (P-to-AP) switching and AP-to-P switching. The characteristic of nonlocal spin injection is analyzed by spin accumulation simulations. The simulation results reveal that spin torque acting on the free layer magnetization does not depend on the magnetization configuration, which explains the smaller asymmetry in *I*_sw_. In addition, we propose the revised device structure in which nonlocal spin injection is expected to be more efficient than direct spin injection.

Here, we comment on how the sample in this study is modified in applications. In the application to an MTJ for MRAM, an additional barrier layer and reference magnetic layer with large magnetoresistance (MR) ratio are required, as shown in Fig. 1c. This type of three-terminal MTJ using an in-plane magnetic material was experimentally demonstrated in ref. ^5^. The write current reverses the free layer magnetization by flowing through the all metallic structure, which can decrease the power consumption. The read current flows in either of the two ways depicted as Read 1 and Read 2 in the figure. By adjusting the barrier layer resistance, unintentional switching of the free layer is prevented, and a large read signal due to the large MR ratio is obtained. In the application to an STO for MAMR, the out-of-plane oscillation of magnetization about the direction perpendicular to the air bearing surface (ABS) can be induced by using an in-plane magnetic material in the free layer, as shown in Fig. 1d. Such oscillation generates a circularly polarized microwave field which can realize magnetization switching solely by a microwave field^27^ or layer selective magnetization switching in a stack of multiple recording layer^28^. Because of the absence of the electrode at the ABS, the free layer can be placed close to the recording media surface. Similar device structure based on SOT was proposed in ref. ^15^.

## Sample Structure and Current Distribution for Direct Spin Injection and Nonlocal Spin Injection

The sample shown in Fig. 1a,b is prepared as follows. We first fabricate a bottom electrode with a contact hole surrounded by insulator 1. The diameter of the contact hole is 150 nm. Subsequently, a continuous Ta seed layer is deposited and planarized. On the flat Ta surface, a giant magnetoresistance (GMR) film consisting of a Co-, Ni-, and Pt-based lower magnetic layer, a 10-nm Cu spacer, and a Co- and Ni-based upper magnetic layer is deposited. The lower and upper magnetic layers are the polarizer and the free layer. The seed layer, polarizer, and spacer are patterned into a mesa of a few micrometers in lateral size, and the free layer is patterned into a nanodot with a diameter of 80 nm on top of the contact hole. Finally, insulator 2 around the free layer and a top electrode are fabricated. Figure 1e shows a cross-sectional transmission electron microscopy image of a sample. The alignment accuracy of the contact hole and the free layer is approximately 25 nm. We make the contact hole larger than the free layer to ensure that the free layer does not deviate from above the contact hole where the spin injection occurs. Figure 1f shows the perpendicular magnetization loop of the GMR film before patterning, which is measured by using a vibrating sample magnetometer (VSM). The polarizer and free layer both have perpendicular magnetization and their magnetization switching is observed when the *z*-direction magnetic field (*H*_*z*_) is ±40 mT and ±25 mT, respectively.

Current application and voltage measurement are conducted through the bottom electrode (BE), the middle electrode (ME) connected to the spacer, and the top electrode (TE). Each electrode has two contact pads on the +*x*- and −*x*-direction sides of the sample, enabling four-probe measurement of the resistance and measurement of the nonlocal spin signal.

**Figure 2:**
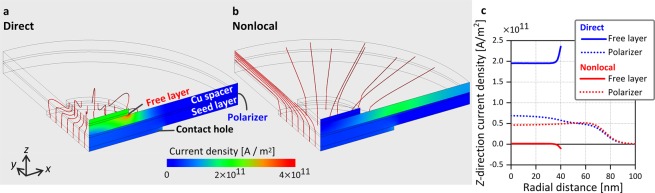
Current distribution. (**a**,**b**) Stream line plot of the computed current distribution for direct and nonlocal spin injection, respectively. Current density is shown on the *x*–*z* plane. (**c**) Computed *z*-direction current density versus radial distance in the middle of the free layer and polarizer.

A current is applied between TE and BE for direct spin injection, and between ME and BE for nonlocal spin injection. We calculate the current distribution for these geometries. In this calculation, we assume that the contact hole and free layer are perfectly aligned and that the current flow is symmetric in the vicinity of the contact hole and free layer because the electrodes and mesa are much larger than the contact hole and free layer. Based on these assumptions, a quarter cylindrical calculation model is used. Figure 2a,b show the computed current distribution for direct and nonlocal spin injection. The current of 0.25 mA is applied in the quarter cylindrical model, which corresponds to 1 mA for the actual device. In direct spin injection, the current density is largest in the spacer near the outer peripheral of the free layer, because the current from the contact hole is concentrated to the smaller free layer. In nonlocal spin injection, the current flows preferentially in the low-resistivity spacer toward its outer peripheral. Figure 2c shows the *z*-direction current density versus the radial distance in the middle of the free layer. In direct spin injection, the current flows almost uniformly in the free layer with a slight increase near the outer peripheral. In nonlocal spin injection, the current density is much smaller than in direct spin injection. More precisely, a small amount of current flows into the free layer near the center and flows out near the outer peripheral. Figure 2c also shows the *z*-direction current density versus the radial distance in the middle of the polarizer. This current brings spin injection from the polarizer to the spacer. Although current through the polarizer is more confined below the free layer in direct spin injection, a similar current amplitude is also realized in nonlocal spin injection. This result indicates that the similar amount of spin injection is expected for both methods.

### Transport measurement

**Figure 3:**
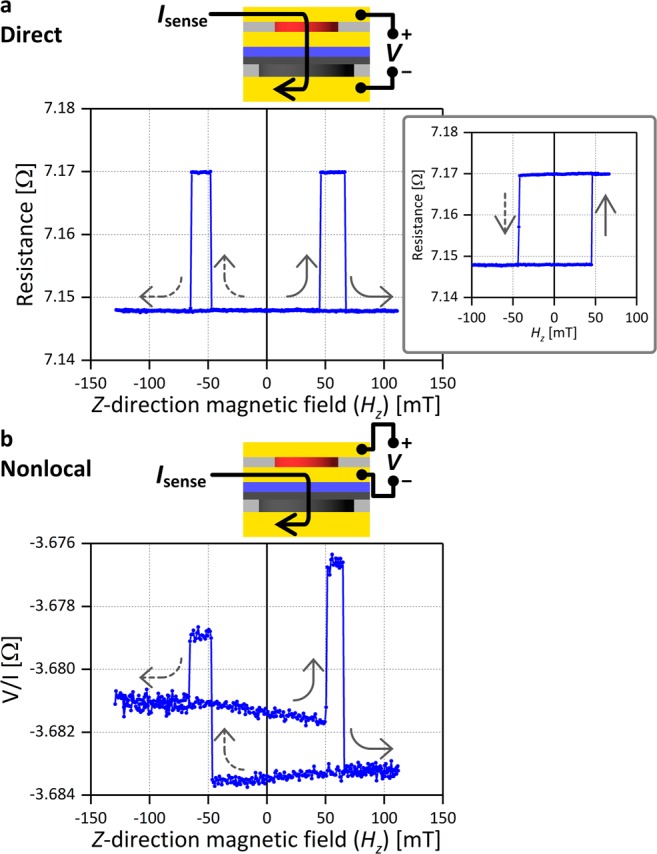
Transport measurement. (**a**) Resistance between BE and TE versus *H*_*z*_. The inset shows the minor loop of free layer switching. (**b**) Nonlocal spin signal versus *H*_*z*_. A schematic of the measurement setup is shown on top of each plot.

We first use the direct spin injection geometry to measure resistance between BE and TE as a function of the *H*_*z*_. Figure 3a shows the result, and the schematic on top shows the measurement setup. An AC sense current (*I*_sense_) of ±100 μA is applied to reduce noise. Sharp transitions at around ±50 mT and ±70 mT correspond to magnetization switching of the free layer and the polarizer, respectively, and two resistance states corresponds to the P and AP states are observed with a resistance difference of 22 mΩ, which is due to the GMR effect. The inset shows the minor loop of free layer switching. The switching field is symmetric, indicating that the stray field from the polarizer is negligible because the polarizer can be regarded as a continuous film in the vicinity of the free layer. The switching fields of the free layer and the polarizer are higher than those of the film sample observed in the VSM data. This is because the free layer and the polarizer are respectively patterned into the nanodot and the mesa, and the patterning reduces nucleation sites. In the free layer, the change of the demagnetizing field due to the patterning also increases the switching field.

Next, we use the nonlocal spin injection geometry by applying current between BE and ME and measuring voltage between ME and TE. The same *I*_sense_ of ±100 μA is applied. Figure 3b shows the measured voltage divided by the current as a function of *H*_*z*_. Similar to the case of direct spin injection, free layer switching occurs at around ±50 mT and changes the spin signal by 5 mΩ. This result indicates that nonlocal spin injection from the polarizer occurs, changing the electrochemical potential between the spacer and the free layer. The amplitude of the spin signal decreases by a factor of four in comparison with that in the case of direct spin injection. According to a previous study^4^, a decrease by a factor of two is explained by the change of the geometry from direct to nonlocal spin injection. The additional decrease is explained by the current distribution; in nonlocal spin injection, the current through the polarizer is less concentrated below the free layer, which decreases the density of the injected spin below the free layer. The change of the spin signal at +70 mT (−70 mT) originating from the polarizer switching is larger (smaller) than that of the free layer switching because it also includes the anomalous Hall effect (AHE) signal of the polarizer. When current from the contact hole flows in the polarizer, it produces AHE voltage in the direction perpendicular to both the current and polarizer magnetization directions. Because the free layer is not perfectly aligned with the contact hole, voltage between ME and TE picks up the AHE voltage of the polarizer. The overall offset of approximately −3.7 Ω is explained as follows. When current flows radially in the vicinity of the contact hole, the current produces a voltage not only in the current-application side of ME (the left side in the Fig. 3b schematic) but also the voltage-measurement (right) side, causing the offset.

### Magnetization switching of the free layer by direct and nonlocal spin injection

**Figure 4:**
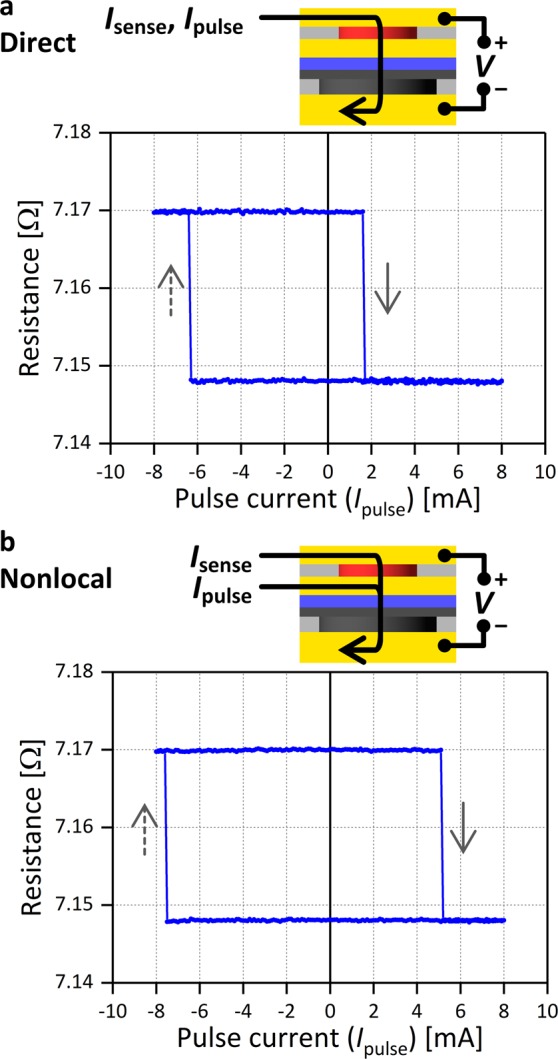
Spin-torque-induced magnetization switching. Sample resistance versus *I*_pulse_ for (**a**) direct spin injection and (**b**) nonlocal spin injection. The sample resistance is measured after applying a single pulse current with a duration of 1 ms.

We measured the spin signal and showed that spin injection from the polarizer occurs in the nonlocal spin injection geometry. We now demonstrate magnetization switching of the free layer by nonlocal spin injection and compare it with that by direct spin injection. Following the order in the previous section, we first show the result of direct spin injection. Figure 4a shows the sample resistance versus a pulse current (*I*_pulse_) for *H*_*z*_ = 0 T. This resistance is measured after applying a single pulse current with a duration of 1 ms. The resistance changes at *I*_pulse_ = + 1.7 mA and −6.4 mA, and the high and low resistance values match those corresponding to the P and AP states observed in the *H*_*z*_ sweeping (Fig. 3a), showing that the free layer magnetization reverses by applying *I*_pulse_. The amplitude of *I*_sw_ for the AP-to-P and P-to-AP switching differs by a factor of almost four. Considering that the stray field from the polarizer is negligible, this asymmetry in *I*_sw_ originates from the asymmetric spin-torque between the P and AP states, which we discuss in the next section using spin accumulation simulations.

We next use the nonlocal spin injection geometry and apply a pulse current between BE and ME. After applying a single current pulse, magnetization switching is detected by measuring resistance between BE and TE. Figure 4b shows the result. Sharp transitions between the P and AP states are observed, showing that magnetization switching of the free layer occurs by nonlocal spin injection from the polarizer. The switching currents are respectively +5.2 mA and −7.6 mA for AP-to-P and P-to-AP switching, and their amplitude differs only by a factor of approximately 1.5. The current amplitude required for magnetization switching is of the same order, and the *I*_sw_ asymmetry is smaller in comparison with the case of direct spin injection. This characteristic of nonlocal spin injection is also discussed in the next section.

Distribution of *I*_sw_ is estimated by repeating the measurement for 100 times, using a different sample with similar *I*_sw_ values. The standard deviation is approximately 0.1 mA, showing that the distribution does not affect the findings from the *I*_sw_ measurements.

### Spin accumulation simulation

**Figure 5:**
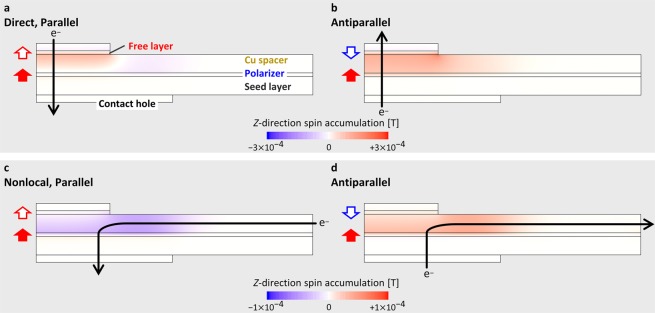
Spin accumulation simulation. Computed *z*-direction spin accumulation for direct spin injection in the initial state of (**a**) P-to-AP switching and (**b**) AP-to-P switching. (**c**,**d**) Corresponding results for nonlocal spin injection.

**Figure 6:**
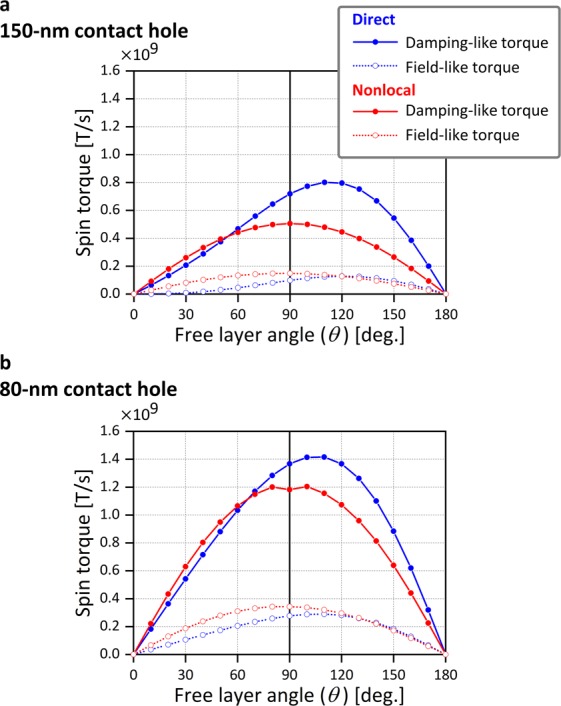
Angle dependence of spin torque. (**a**) Computed spin torque versus the free layer magnetization direction for the model with the 150-nm contact hole. (**b**) Corresponding results for the model with the 80-nm contact hole.

This section describes spin accumulation simulations for analyzing the experimental data. We first calculate the spatial distribution of spin accumulation, and then calculate spin torque acting on the free layer. Note the following about the simulations. The diffusion constant of 10^−4^ m^2^/s is tentatively employed for all materials because we were unable to find the values in the literature. Considering that spin accumulation is inversely proportional to the diffusion constant, the spatial distributions of spin accumulation shown in Fig. 5a–d are the qualitative comparison between direct and nonlocal spin injection. However, because spin torque is proportional to both spin accumulation and diffusion constant, the computed spin torques shown in Fig. 6a,b do not depend on the diffusion constant.

Figure 5a,b show a color plot of *z*-direction spin accumulation in the case of direct spin injection for the initial state of P-to-AP and AP-to-P switching. The current of 0.25 mA is applied in the quarter cylindrical model, corresponding to 1 mA in the actual device. Because current flows through both free layer and polarizer, current is spin-polarized by both layers. As a result, the spin accumulation introduced to the spacer from the two magnetic layer cancels out for the P state, and is enhanced for the AP state. Because both magnetic layers contribute to spin accumulation in a constructive or destructive way, spin accumulation strongly depends on the magnetization configuration.

Figure 5c,d show the corresponding simulation results for nonlocal spin injection. Spin accumulation is symmetric for the P and AP states because current flows through only the polarizer. It is also found that the spin accumulation occurs in the broader area than in direct spin injection because of the spread current distribution. The spin accumulation in the interface between the spacer and the polarizer is almost uniform above the contact hole, reflecting the fact that the current density in the polarizer is almost uniform in this region (Fig. 2c). This result indicates that the lateral shift of the free layer from the center of the contact hole has little effect unless the free layer deviates from above the contact hole.

We next calculate spin torque acting on the free layer from the result of the spin accumulation simulation. To discuss the relation between spin torque and magnetization switching, we consider the Landau–Lifshitz–Gilbert equation, which describes the dynamics of the free layer magnetization (**M**^free^) as

1 $$\frac{d{{\bf{M}}}^{{\rm{free}}}}{dt}=-|\gamma |{{\bf{M}}}^{{\rm{free}}}\times {{\bf{H}}}_{{\rm{eff}}}^{{\rm{free}}}+\alpha \frac{{{\bf{M}}}^{{\rm{free}}}}{{M}^{{\rm{free}}}}\times \frac{d{{\bf{M}}}^{{\rm{free}}}}{dt}+{\bf{T}}.$$

here, *γ* denotes the gyromagnetic ratio and *α* denotes the damping constant. The effective field acting on the free layer (${{\bf{H}}}_{{\rm{eff}}}^{{\rm{free}}}$) consists of the anisotropy field and demagnetizing field, and **T** denotes the average spin torque of the all the cells in the free layer. For simplicity, we assume the uniform magnetization in the free layer and fix the polarizer magnetization in the + *z* direction. Using the two unit vectors of the free layer magnetization and the polarizer magnetization directions (**m**^free^ and **m**^polarizer^), **T** is decomposed into two components as

2 $${\bf{T}}=-{T}_{{\rm{f}}}{{\bf{m}}}^{{\rm{free}}}\times {{\bf{m}}}^{{\rm{polarizer}}}-{T}_{{\rm{d}}}{{\bf{m}}}^{{\rm{free}}}\times ({{\bf{m}}}^{{\rm{free}}}\times {{\bf{m}}}^{{\rm{polarizer}}}).$$

The first term on the right-hand side is a field-like torque and the second term is a damping-like torque. In our device, ${{\bf{H}}}_{{\rm{eff}}}^{{\rm{free}}}$ and **m**^polarizer^ are in the perpendicular direction, and the damping-like torque mainly contributes to magnetization switching by overcoming the damping term (the second term on the right-hand side in Eq. (1)). The magnitude of the two vectors **m**^free^ × **m**^polarizer^ and **m**^free^ × (**m**^free^ × **m**^polarizer^) is sin*θ*, where *θ* is the angle of free layer magnetization with respect to the +*z* direction. Spin torques including this magnitude of the vectors, namely *T*_*f*_ sin*θ* and *T*_*d*_ sin*θ*, are shown in Fig. 6a as a function of *θ*. No spin torque acts on the free layer for the collinear configurations at 0° and 180°. This means that magnetization switching requires thermal fluctuation of the magnetization to create an initial angle. The calculation is performed for positive current and yields positive *T*_*d*_. The positive *T*_*d*_ aligns the free layer magnetization toward the polarizer magnetization direction, corresponding to AP-to-P switching. For P-to-AP switching, the current is reversed. However, current reversal only changes the sign of *T*_*d*_. Because ${{\bf{H}}}_{{\rm{eff}}}^{{\rm{free}}}$ is largest at 0° and 180°, *I*_sw_ values for P-to-AP and AP-to-P switching are respectively determined by the absolute values of *T*_*d*_ sin*θ* near 0° and 180°. In the case of direct spin injection, *T*_*d*_ depends on *θ* because both free layer and polarizer contribute to the spin injection, yielding the asymmetric *T*_*d*_ sin*θ*, in which the spin torque is stronger in the AP state. The *T*_*d*_ sin*θ* value at 170° is larger than that at 10° by a factor of three, which roughly agrees with the four-fold *I*_sw_ asymmetry observed in the experiments. In the case of nonlocal spin injection, *T*_*d*_ depends little on the free layer magnetization angle, yielding symmetric and sinusoidal *T*_*d*_ sin*θ*. The amplitude of the spin torque is of the same order as that for direct spin injection. This result is consistent with the experimental result where *I*_sw_ is of the same order and shows smaller asymmetry.

### Improvement of device structure and advantage of nonlocal spin injection in spintronic applications

Overall, the experimental results are explained by the spin accumulation simulations. However, the following disagreement exists. The simulation result in Fig. 6a shows larger spin torque around the P state for nonlocal spin injection, which means that *I*_sw_ for P-to-AP switching is expected to be smaller for nonlocal spin injection than for direct spin injection. It is, however, slightly larger in the experiments. This disagreement can be explained as follows. When the upper magnetic layer is patterned into the nanodot by Ar ion milling, the spacer layer is damaged, and mixing with the other materials occurs. In addition, the spacer is exposed to air before insulator 2 is fabricated. As a result, the spin transport property of the spacer outside of the free layer might deteriorate, which is not considered in the simulations. In nonlocal spin injection, the spin injection is less confined below the free layer. Thus, the spin injected around the free layer attenuates because of the damage in the spacer, deteriorating spin injection efficiency. One design to solve this problem is to make the contact hole as small as or smaller than the free layer and to align them as close as possible. By this design change, spin injection is more confined below the free layer, where the spacer is protected by the free layer and is undamaged. In addition, confined spin injection increases spin-torque efficiency. As an example, we use the model in which both free layer and contact hole have a diameter of 80 nm and calculate the spin torque, as shown in Fig. 6b. The current is set to 1 mA in the actual device, which is same as that in Fig. 6a. By using a smaller contact hole, the spin torque increases for both direct and nonlocal spin injection. In the case of the 150-nm contact hole, the damping torque at 90° for direct spin injection is 50% more efficient than that for nonlocal spin injection. This difference narrows to 15% in the case of the 80-nm contact hole. This is because the direct spin injection geometry no longer benefits from current confinement by the free layer as it is already confined by the small contact hole. By making the contact hole small, nonlocal spin injection can benefit from smaller *I*_sw_ for P-to-AP switching because this structure circumvents the effect of the damaged spacer.

Symmetric spin torque of nonlocal spin injection is advantageous in the following applications. In MRAM, the write current circuit needs to take care of the *I*_sw_ asymmetry and is capable of generating the larger *I*_sw_ for P-to-AP switching^29^. In this respect, the current capability can be mitigated when the *I*_sw_ for P-to-AP switching reduces in nonlocal spin injection, as expected from the simulation on the revised device structure. This kind of spin torque can also reduce operation current in the type of STO proposed for MAMR^23^. In the STO operation, the two magnetic layers of the STO are first in the P state owing to the gap field of the write head. By applying a current, the magnetic oscillation layer starts to reverse, leading to magnetization oscillation. The larger spin torque near the P state can thus reduce operation current.

## Conclusions

We experimentally demonstrated the magnetization switching of a perpendicular magnetic nanodot induced by injection of pure spin current using a nonlocal geometry. Because perpendicular spin is injected when a perpendicular magnetic material is employed as a polarizer, the magnetization switching requires no additional assist such as an external field, which is one of the advantages of nonlocal spin injection over SOT. In comparison with magnetization switching by direct spin injection, *I*_sw_ is of the same order and shows smaller asymmetry between P-to-AP and AP-to-P switching. Spin accumulation simulation shows that spin torque does not depend on the magnetization configuration, which explains the small *I*_sw_ asymmetry. This characteristic of nonlocal spin injection can be advantageous in the application such as MRAM and the STO for MAMR, in which symmetric spin torque or spin torque at small angles is crucial.

## Methods

### Sample fabrication

A Ta (5)/Cu (40)/Ta (10) bottom electrode is sputter-deposited on a sapphire substrate. (Here and below, numbers in parentheses are thicknesses in nm.) A pillar with a diameter of 150 nm is fabricated by electron beam lithography and Ar ion milling, followed by deposition of a thin Ta barrier layer and SiO_2_ insulator (insulator 1) and lift-off. A Ta (40) seed layer is sputter deposited and planarized by chemical-mechanical planarization, leaving approximately 10 nm of the seed layer. On the Ta surface, a perpendicular GMR film is deposited by using a magnetron sputtering system. The film structure consists of the following layers: Ta (2)/Pt (3)/Co (0.27)/Pt (0.5)/[Co (0.27)/Ni (0.6)] × 2/Co (0.27)/Cu (10)/[Co (0.27)/Ni (0.6)] × 2/Co (0.27)/Pt (1)/Ta (1)/Ru (3). A free layer with a diameter of 80 nm is fabricated by electron beam lithography and Ar ion milling, followed by deposition of a SiO_2_ insulator (insulator 2) and lift-off. Finally, a Ti (10)/Au (40) top electrode is fabricated.

### Simulation

The simulations of current distribution and spin accumulation are conducted using the same quarter-cylindrical model. The model consists of a contact hole (4)/seed layer (10)/polarizer (2)/spacer (10)/free layer (2)/cap layer (4). The radius of the contact hole is 75 or 40 nm, that of the seed layer, polarizer, and spacer is 150 nm, and that of the free layer and cap layer is 40 nm. The model is discretized by using tetrahedral cells with average size of 1 nm in the magnetic layers and 2 nm in the other layers. We employ the physical properties of Co/Ni multilayer, Cu, and Ta for the magnetic layers, spacer, and other layers, respectively. The current distribution is calculated by using the finite-element software JMAG. The resistivity for Co/Ni, Cu, and Ta is set to 2.5 × 10^−7^, 5 × 10^−8^, and 2 × 10^−6^ Ω·m, respectively. The value of Ta is that of β-phase Ta. During sample fabrication, we measured the sheet resistance of the Ta seed layer and obtained 2.2 × 10^−6^ Ω·m, which agrees with the simulation parameters. The spin accumulation is calculated by using the finite-element simulation code based on the theory presented in ref. ^30^ and developed by our group. The parameters are as follows. The spin diffusion length for Cu and Ta is 300 and 2 nm. The longitudinal spin diffusion length, transverse spin diffusion length, and spin polarization for Co/Ni multilayer are 5 nm, 1 nm, and 0.6, respectively^31,32^. A diffusion constant of 10^−4^ m^2^/s is tentatively employed for all materials.

## Data Availability

The datasets generated during and/or analyzed during the current study are available from the corresponding author upon reasonable request.
